# Supplementary material for: High and stable ATP levels prevent aberrant intracellular protein aggregation in yeast
Source: eLife. 2022 Apr 19;11:e67659. doi: 10.7554/eLife.67659 (PMC9018071; doi:10.7554/eLife.67659)
Supplement: Supplementary file 1. [file elife-67659-supp1.docx]

## Table S1. *Strains used in the present study*

| Name | Genotype | Source | Figure |  |
| --- | --- | --- | --- | --- |
| MTY3008 | *snf1∆::kanMX6 leu2Δ0 lys2Δ0 ura3Δ0* | Lab stock | 1C, 3B, 5A-C, 5E, Figure 5—figure supplement 1 |  |
| MTY3015 | *his3Δ1 leu2Δ0 lys2Δ0 ura3Δ0* | Lab stock | 1C, 1F, 3B, 5A-E, Figure 5—figure supplement 1 |  |
| MTY3049 | *adk1∆::kanMX6 his3Δ1 leu2Δ0 lys2Δ0 ura3Δ0* | Lab stock | 1C, 3B, 5A-E, Figure 5—figure supplement 1 |  |
| MTY3118 | *bas1∆::kanMX6 leu2Δ0 lys2Δ0 ura3Δ0* | Lab stock | 1F, 3B, 5A-E, Figure 5—figure supplement 1 |  |
| MTY3143 | *his3Δ1:: 2×pRS303-P_TEF_-QUEEN-2m-T_CYC1_ leu2Δ0 lys2Δ0 ura3Δ0* | This study | Figure 1—figure supplement 2 |  |
| MTY3149 | *adk1∆::kanMX6 his3Δ1:: 2×pRS303-P_TEF_-QUEEN-2m-T_CYC1_ leu2Δ0 lys2Δ0 ura3Δ0* | This study | Figure 1—figure supplement 2 |  |
| MTY3264 | *his3Δ1:: 3×pRS303-P_TEF_-QUEEN-2m-T_CYC1_ leu2Δ0 lys2Δ0 ura3Δ0 MYO1-3mCherry-hphMX6* | Takaine et al., 2019 | 1A-B, 1E, 2A, 2F, Figure 1—figure supplement 1, Figure 1—figure supplement 4, Figure 2—figure supplement 2A |  |
| MTY3270 | *bas1∆::kanMX6 his3Δ1:: 3×pRS303-P_TEF_-QUEEN-2m-T_CYC1_ leu2Δ0 lys2Δ0 ura3Δ0 MYO1-3mCherry-hphMX6* | This study | 1D-E, 2G, Figure 1—figure supplement 4, S7 |  |
| MTY3293 | *adk1∆::kanMX6 his3Δ1:: 3×pRS303-P_TEF_-QUEEN-2m-T_CYC1_ leu2Δ0 lys2Δ0 ura3Δ0 MYO1-3mCherry-hphMX6* | This study | 1A-B |  |
| MTY3355 | *adk1∆::kanMX6 snf1∆::natNT2 his3Δ1:: 3×pRS303-P_TEF_-QUEEN-2m-T_CYC1_ leu2Δ0 lys2Δ0 ura3Δ0 MYO1-3mCherry-hphMX6* | This study | 1A-B, 2B-F, Figure 2—figure supplement 1, Figure 2—figure supplement 2B-C |  |
| MTY3371 | *snf1∆::kanMX6 his3Δ1:: 3×pRS303-P_TEF_-QUEEN-2m-T_CYC1_ leu2Δ0 lys2Δ0 ura3Δ0 MYO1-3mCherry-hphMX6* | This study | 1A-B, Figure 1—figure supplement 1, Figure 1—figure supplement 4 |  |
| MTY3412 | *adk1∆::kanMX6 snf1∆::natNT2 leu2Δ0 lys2Δ0 ura3Δ0* | This study | 1C, 3B, 5A-E, Figure 5—figure supplement 1 |  |
| MTY3401 | *gal83∆::kanMX6 his3Δ1:: 3×pRS303-P_TEF_-QUEEN-2m-T_CYC1_ leu2Δ0 lys2Δ0 ura3Δ0 MYO1-3mCherry-hphMX6* | This study | Figure 1—figure supplement 1C | |
| MTY3403 | *sip1∆::kanMX6 his3Δ1:: 3×pRS303-P_TEF_-QUEEN-2m-T_CYC1_ leu2Δ0 lys2Δ0 ura3Δ0 MYO1-3mCherry-hphMX6* | This study | Figure 1—figure supplement 1C | |
| MTY3405 | *sip2∆::kanMX6 his3Δ1:: 3×pRS303-P_TEF_-QUEEN-2m-T_CYC1_ leu2Δ0 lys2Δ0 ura3Δ0 MYO1-3mCherry-hphMX6* | This study | Figure 1—figure supplement 1C | |
| MTY3417 | *mig1∆::natNT2 his3Δ1:: 3×pRS303-P_TEF_-QUEEN-2m-T_CYC1_ leu2Δ0 lys2Δ0 ura3Δ0 MYO1-3mCherry-hphMX6* | This study | Figure 1—figure supplement 1D |  |
| MTY3420 | *Hsp104-yeGFP-hphNT1 his3Δ1 leu2Δ0 lys2Δ0 ura3Δ0* | This study | 3C-D, 4A-B |  |
| MTY3421 | *snf1∆::kanMX6 Hsp104-yeGFP-hphNT1 his3Δ1 leu2Δ0 lys2Δ0 ura3Δ0* | This study | 3C-D |  |
| MTY3422 | *bas1∆::kanMX6 Hsp104-yeGFP-hphNT1 his3Δ1 leu2Δ0 lys2Δ0 ura3Δ0* | This study | 3C-D |  |
| MTY3424 | *adk1∆::kanMX6 Hsp104-yeGFP-hphNT1 his3Δ1 leu2Δ0 lys2Δ0 ura3Δ0* | This study | 3C-D |  |
| MTY3425 | *adk1∆::kanMX6 snf1∆::natNT2 Hsp104-yeGFP-hphNT1 his3Δ1 leu2Δ0 lys2Δ0 ura3Δ0* | This study | 3C-D |  |
| MTY3489 | *atg1∆::kanMX6 his3Δ1 leu2Δ0 lys2Δ0 ura3Δ0* | Lab stock | 5E |  |
| MTY3493 | *snq2∆::KILeu2; pdr3∆::KIura3; pdr1∆::natMX4; can1∆::STE2prSp_his5 lyp1∆ his3∆1 leu2∆0 ura3∆0 met15∆0 LYS2+* | From Y. Ohya, ([Piotrowski *et al.*, 2017](#_ENREF_45)) | Parental strain of MTY3501 |  |
| MTY3501 | *Hsp104-yeGFP-hphNT1 snq2∆::KILeu2; pdr3∆::KIura3; pdr1∆::natMX4; can1∆::STE2prSp_his5 lyp1∆ his3∆1 leu2∆0 ura3∆0 met15∆0 LYS2+* | This study | 5F |  |
| MTY3503 | *Pab1- yeGFP-hphNT1 his3Δ1 leu2Δ0 lys2Δ0 ura3Δ0* | This study | 3D |  |
| MTY3504 | *Hsp104-yeGFP-hphNT1 Pab1-RedStar2-natNT2 his3Δ1 leu2Δ0 lys2Δ0 ura3Δ0* | This study | Figure 4—figure supplement 1 |  |
| MTY3505 | *snf1∆::kanMX6 Pab1- yeGFP-hphNT1 his3Δ1 leu2Δ0 lys2Δ0 ura3Δ0* | This study | 3D |  |
| MTY3506 | *adk1∆::kanMX6 Pab1- yeGFP-hphNT1 his3Δ1 leu2Δ0 lys2Δ0 ura3Δ0* | This study | 3D |  |
| MTY3507 | *adk1∆::kanMX6 snf1∆::natNT2 Pab1- yeGFP-hphNT1 his3Δ1 leu2Δ0 lys2Δ0 ura3Δ0* | This study | 3D |  |
| MTY3508 | *bas1∆::kanMX6 Pab1- yeGFP-hphNT1 his3Δ1 leu2Δ0 lys2Δ0 ura3Δ0* | This study | 3D |  |
| MTY3513 | *atg1∆::natNT2 adk1∆::kanMX6 his3Δ1 leu2Δ0 lys2Δ0 ura3Δ0* | This study | 5E |  |
| MTY3514 | *atg1∆::natNT2 snf1∆::kanMX6 his3Δ1 leu2Δ0 lys2Δ0 ura3Δ0* | This study | 5E |  |
| MTY3515 | *atg1∆::natNT2 bas1∆::kanMX6 his3Δ1 leu2Δ0 lys2Δ0 ura3Δ0* | This study | 5E |  |
| MTY3516 | *rpn4∆::natNT2 adk1∆::kanMX6 his3Δ1 leu2Δ0 lys2Δ0 ura3Δ0* | This study | 5E |  |
| MTY3517 | *rpn4∆::natNT2 snf1∆::kanMX6 his3Δ1 leu2Δ0 lys2Δ0 ura3Δ0* | This study | 5E |  |
| MTY3518 | *rpn4∆::natNT2 bas1∆::kanMX6 his3Δ1 leu2Δ0 lys2Δ0 ura3Δ0* | This study | 5E |  |
| MTY3525 | *rpn4∆::kanMX6 his3Δ1 leu2Δ0 lys2Δ0 ura3Δ0* | Lab stock | 5A-C, 5E |  |
| MTY3839 | *his3Δ1:: 3×pRS303-P_TEF_-QUEEN-2m-T_CYC1_ leu2Δ0 ura3Δ0 Hsp104-RedStar2-natNT2* | This study | 6A-D, Figure 6—figure supplement 1 | |
| MTY3845 | *adk1∆::kanMX6* *snf1∆::hphNT1* *his3Δ1:: 3×pRS303-P_TEF_-QUEEN-2m-T_CYC1_ leu2Δ0 ura3Δ0 Hsp104-RedStar2-natNT2* | This study | 6E-F, 7, Figure 6—figure supplement 2, Figure 7—figure supplement 1-2 | |
