# Supplementary material for: High and stable ATP levels prevent aberrant intracellular protein aggregation in yeast
Source: eLife. 2022 Apr 19;11:e67659. doi: 10.7554/eLife.67659 (PMC9018071; doi:10.7554/eLife.67659)
Supplement: Supplementary file 2. [file elife-67659-supp2.docx]

## Table S2. *Plasmids used in the present study*

| Name | Structure | Source | Purpose |
| --- | --- | --- | --- |
| MTP3091 | pESC-Leu-GFP-Ubc9-ts | Judith Frydman (pJF1089) | Figure 4C |
| MTP3088 | p426 103Q GAL | Addgene (cat.# 1188) | Figure 5—figure supplement 1 |
| MTP3108 | pYES2-α-synuclein-GFP | H. Takagi ([Wijayanti *et al.*, 2015](#_ENREF_58)) | Figure 5A-D |
| MTP3090 | pYES2-GFP | K. Ohashi | Figure 5A |
